# Hallmarks of disease: how tuned hierarchies of intelligent molecular neural networks (MNNs) may matter

**DOI:** 10.1530/ERC-25-0183

**Published:** 2026-01-05

**Authors:** Hans V Westerhoff

**Affiliations:** ^1^ Department of Molecular Cell Biology, Amsterdam Institute for Molecules, Medicines and Systems, Vrije Universiteit Amsterdam, Amsterdam, The Netherlands; ^2^ School of Biological Sciences, Faculty of Biology, Medicine and Health, The University of Manchester, Manchester, UK; ^3^ Swammerdam Institute for Life Sciences, University of Amsterdam, Amsterdam, The Netherlands; ^4^ Stellenbosch Institute for Advanced Study (STIAS), Wallenberg Research Centre at Stellenbosch University, Stellenbosch, South Africa

**Keywords:** systems biology, network principles, molecular neural networks, network diseases, artificial intelligence

## Abstract

The intracellular molecular networks are highly complex and plastic. Yet, they obey the network principles discovered by systems biology. Precise mathematical models of some networks enable one to predict how molecular properties determine the function and malfunction of the network. However, the complexity is even greater than this: due to selection in evolutionary biology, the molecular networks are not only causal to cell function, but also caused by the requirement that the cell’s functions be optimal. We discuss how the combination of complexity, plasticity, and this circular causality makes the networks similar to trained artificial neural networks. Causation by purpose might thereby dominate over causation by mechanism. Comparison of challenges that may cause or cure disease with actual challenges in evolutionary, developmental, or cell biology, and assessment of the learned responses thereto, might add another interesting layer of systems biology.

## Molecules and networks

Molecular biochemistry has been tremendously successful in unraveling mechanisms by which macromolecules such as nucleic acids and proteins work. Major steps include the determination of detailed structures of nucleic acids and proteins, the cloning of DNA fragments corresponding to genes, the transfection of living cells with such genes, the site-directed insertion of novel genes into the genomes of living cells, the precise modulation of regulatory DNA sequences *in situ*, and the precise modulation of protein activities by site-directed mutagenesis.

In the analogy of a car, we now know the structure and action mechanism of many of the parts, such as the steering wheel, the cylinders, and the spark plugs, and we know that they function; e.g., by pressing the accelerator, we change the firing frequency of the spark plugs and the frequency by which the wheels rotate, and this helps us drive the car up a steeper slope of the motorway. However, few of us understand how all these parts work together, i.e., how a change in battery power may affect the frequency at which the spark plugs fire, and how this affects our highway speed and acceleration. If the car breaks down, we are unable to fix it. And when we bring it to the automotive workshop, the technicians can only fix it by using complex computer software to repair, or more often, just replace a well-defined component with a new copy with identical functional (input–output) specifications. Car manufacturers and repair shops use computer programs in which all car components’ interactions with other components are captured. By numerical integration, the effects of failure of a component on the car’s performance can be predicted. And retroactively, if the car fails to perform a certain function, one may calculate what the most likely failures of parts of the car are. Mathematical modeling can help us understand the linkages between component properties and the functioning of the whole.

Already a while ago, we (and many others) embarked on this insight and made ‘Swiss watch’/‘silicon cell’ models of biochemical networks that manage health and disease (1). One series of these models helped us identify an unexpected target for drugs that might help kill *Trypanosoma brucei*, the causative organism of sleeping sickness (2). Another series helped us understand the functioning of the networks around ROS (reactive oxygen species) signaling (3), which more recently culminated in new insights into how mitochondrial suicide (mitoptosis) may function in neural differentiation (4). The scientific endeavor of understanding the network (rather than component) mechanisms that help cause disease and health is called systems biology/systems medicine. The workhorses in systems biology (5) are mathematical modeling, precise experimental mining and assessment of component properties, and then validation of predictions in more physiological experiments (6, 7). When addressing genome-wide systems, systems biology involves bioinformatics to recognize patterns in the data. With the vast accumulation of biological and medical data, this activity has come to be assisted by data science, where machine learning has been helpful. Artificial intelligence may soon be helpful as well, even though the aim of systems biology is mechanistic understanding rather than understanding by an artificial neural network (8).

## Component versus network principles

Biology is highly diverse. Yet, studies of its macromolecules have revealed a number of general principles. Examples include the fact that DNA tends to occur as a double helix, that protein structures contain alpha helices and beta sheets and have tertiary structures that are determined by interactions by rest groups of amino acids that are distant in terms of the primary structure, and that reaction rates of enzymes have a maximum. Biochemical reactions can only run downhill in terms of apparent Gibbs energy (9). Virtually all reactions are catalyzed by proteins.

Biochemical networks also come with principles (e.g., (3, 10, 11, 12, 13, 14, 15, 16, 17, 18, 19, 20, 21, 22)) and systems biology has helped to identify them. Metabolic fluxes involve networks of enzymes and have maximal values. The actual steady-state flux is, however, controlled by multiple enzymes in the pathway, as are the concentrations of the intermediary metabolites. Enzymes that are highly responsive to changes in the concentrations of their substrates or products exercise little control over the flux. Amplification of the concentration of an enzyme reduces its control over the flux.

These principles have been shown to be properties already of simple, linear biochemical networks. They were thereby readily shown by simulations using the watchmaker models, experimentally, and even theoretically/mathematically (21). They therefore seem to be inescapable.

## Biology

Living organisms are confronted with multiple challenges. Some of these challenges have been met by the evolution of enzymes that are able to catalyze reactions much faster than they happen in dead nature. Thereby, desired reactions proceed much faster than undesired ‘dead’ reactions. The challenge of complex chemistry, where a molecule (e.g., glucose) has to undergo multiple chemical transformations before it is useful as a building block (e.g., alanine) for a biological structure, has been met by the evolution of multiple enzymes, each carrying out one of the required reactions, and the evolution of a membrane keeping the chemical intermediates at sizable concentrations. The challenge that some important biochemical processes, such as the assimilation of CO_2_ and ammonia, are uphill thermodynamically and thereby in themselves impossible has been addressed by merging a protein catalyzing the uphill reaction with a protein catalyzing a thermodynamically downstream reaction in such a way that the two processes became coupled (9). In situations where important metabolic fluxes are limited by a *V_max_* (23), hierarchical regulation has evolved, meaning that the gene encoding the limiting enzyme became more highly expressed (11).

## Phosphoneural networks and intelligence

In the four billion years of biological evolution and its more than a trillion cell cycles with mutations, molecules and networks produced have been selected for the fitness they conferred on the organism. It was advantageous to sense the advent of a nutrient earlier than competing organisms did, and hence a protein emerged that could position itself stably in the cell’s lipid bilayer and detect the molecule already at low concentrations. The evolution of active transport then enabled enzymes metabolizing the nutrient at higher concentrations to catalyze the metabolism at relevant high rates. In order to detect the molecule at even lower concentrations, additional networks evolved that were able to amplify the signal. Important functions such as growth require the presence of diverse nutrients at the same time. Hence, signal transduction and gene expression networks have evolved that respond in logical ways to lack of some nutrients, shortage of the chemical elements CHNOPSMg, and other possible impediments to growth. Accordingly, most living cells are host to proteinaceous networks that enable a kind of molecular intelligence (24, 25).

## Whatever it takes

It appears that lengthy evolution has enabled living organisms to develop ‘whatever it takes’ to meet important challenges to their fitness. This comprises the evolution of extremely complex networks. The complexity of regulation of metabolism may well be much higher than the complexity of metabolism itself. At any rate, the actual organization of living organisms extends far beyond the minimum required to meet the principle of Occam’s razor in its usual implementation, i.e., to explain phenomena by the simplest hypothesis possible (17, 19).

This evolutionary history of living systems has had at least three consequences. First, the resulting molecules and networks are extremely complex. Indeed, enzymes have highly complex structures, and biochemical networks are highly complex in terms of their organization (26). This has provided organisms with amazing abilities to perform their tasks fast and when required. The second consequence is that the networks are robust with respect to the multiple challenges to which they have been exposed during evolution, including mutagenesis. To this end, most routes through metabolic networks have backup routes, and most genes appear to be redundant (12). The third consequence is that the molecules and networks found in living systems do not need to be the very best possible *vis-à-vis* their function. In evolution, organisms can have undergone a mutation that improved fitness but led into an evolutionary dead-end street, so that further optimization through evolution was impossible. Assuming complete optimality may therefore be too simple in many cases.

## Circular causality and dual understanding

Changes in the network and/or the molecules therein cause the organism to change its function in a certain way. This is an example of ‘efficient causation’, as defined by Aristotle. Through biological evolution, the networks and molecules of living cells have in turn been caused by the required fitness. This is so-called final (teleological) causation, i.e., causation by purpose. This second type of causation is or should be alien to physical and chemical systems. It is relevant in technological systems, however, where mechanisms have been designed by human intelligence in order to be capable of fulfilling certain functions. It is also relevant in evolutionary biology, as noted already by Darwin (27). The combination of efficient and final causation constitutes circular, or perhaps rather ‘spiraling’ causation, in which the networks have caused themselves by causing function that then causes the network structure to evolve to an improved function. By distinguishing between actual (minutes) and evolutionary time scales (millennia), some circular causation can be disentangled. Other types of circular causation in biology can be more difficult to disentangle, e.g., that of the lactose level inside a cell causing the expression of the lactose permease, which causes, in real time, a change in the level of intracellular lactose. Here, rapid experiments or mechanistic analyses and modeling are required to disentangle the two causalities (15).

Final causation plays a role in determining the growth rates of microorganisms such as yeast and *E. coli*. An increase in the membrane concentration of the proton-translocating ATPase in the latter should cause the growth rate to increase in a bout of efficient causation. Yet, because the expression of the ATPase genes competes with the expression of genes encoding ribosomes, the growth rate might actually decrease through a different route of efficient causation. There is a trade-off between increased concentrations of proteins enhancing growth rate and increased cost of protein synthesis retarding it, and this in the context of membranes and ribosomes. This may explain why the actual concentration of the proton-translocating ATPase is optimal with respect to the specific growth rate (28): because evolution altered the expression of the ATPase genes (and other genes) until the optimal trade-off giving the maximum specific growth rate was reached. This means that the concentration of the ATPase (and other proteins) is caused teleologically by the specific growth rate ‘aiming’ to be maximal (22), an example of final causation. The cellular composition and distribution over elementary flux modes predicted by a flux balance analysis (FBA) with maximum specific growth rate as objective, and hence accommodating final causation, are close to what is observed experimentally (29), suggesting that we are close to understanding the final causation here. It is important here that the cell-cycle time of *E. coli* is short, so that most cultures grown for weeks under conditions selecting for maximum specific growth rate may have come close to the optimal state (30).

Should the concentration of the proton-translocating ATPase drop below its optimum concentration due to a mutation in its promoter, the specific growth rate will be lower than the maximal growth rate and will not automatically, at the time scale of the cell cycle, increase to that maximum. A new set of mutations and Darwinian selection for the highest specific growth rate will be needed for that, indeed a new round of evolution. This type of final causation requires evolutionary time scales because the mechanism requires the generation of sustained (genetics-based) alternatives followed by selection for the function that is the aim of the final causation. For mammalian cell biology with primary cell cultures, one may therefore expect the expression pattern of the proteins to correspond to a distribution that is optimal for the functioning of the cells in the tissue from which they originate. However, upon a perturbation, e.g., due to a gene mutation or an unusual change in experimental conditions such as temperature or an unusual metabolic substrate, the cell’s protein network will not be optimal for that function.

Because of the long cell-cycle time, the evolution of the cells to the optimal protein expression state will be much too slow to witness. There are two exceptions to this. One is where the cell population was heterogeneous to start with. Then, the subpopulations with the optimal protein distribution may take over within a few cell cycles. The other is that of cell populations that have undergone somatic mutations that greatly enhance the mutation rates. This will reduce the time scale at which evolution occurs. This may allow selection against growth rate suppression by growth factors (31) or selection for robustness with respect to limited oxygen supply through a Warburg-type effect (32).

Metabolic networks are amenable to FBA because all their processes are connected by the flow of chemical mass. Gene expression networks such as those leading from DNA to mRNA to proteins to metabolites are ‘hierarchical’; they are not connected by mass flow. DNA neither becomes mRNA nor noncoding RNA, and neither of these becomes protein. Signal transduction networks such as the MAP kinase cascade are similarly ‘hierarchical’ (33). That the networks of living cells are hierarchical is yet another complication. It leads to even more sites of control, to amplification (e.g., one mRNA leading to multiple copies of the corresponding protein), and to a diversification of time scales. This complexity may explain why, after decennia of scientific research, the physiological functioning of living organisms and their cells is still ill understood (18).

The parallel existence of efficient and final causation in biology reinforces that cause–effect relationships play roles also in living organisms. Life is more than big data; it is also the cause–effect relations between the data. Systems biology’s understanding of these relationships is causal and related to the ‘do calculus’ proposed by Pearl (34) to remediate the lack of causality in data science and Bayesian approaches, where it is replaced by mere statistical correlations.

The phosphoneural networks, intelligence, and circular causality are aspects of biological networks that are parts of the complexity of the latter. Where complexity has long been defined more loosely as that which brings emergence of new properties through nonlinearities, such as in H_2_O as compared to H_2_ plus O_2_ (14, 17, 19), it has recently been defined more precisely (20).

## Disease

If understanding physiological function is so challenging, how well can we expect diseases to be understood? The inborn errors of metabolism constitute an interesting case. Many of these have been linked to mutations in single genes. For phenylketonuria (PKU), this is the gene encoding phenylalanine hydroxylase. Changes in this gene are thereby considered to be the single cause of the disease of most PKU patients. Still, it was not immediately clear why provision of the product of phenylalanine hydroxylase, i.e., tyrosine, to the patient does not cure the disease, whereas a phenylalanine-free diet does. This issue has recently been resolved by a new FBA method, which is able to deal with competition between metabolites for transport (35). This study further revealed that the inactivation of tyrosine hydroxylase did not only affect protein synthesis, but also the synthesis of dopamine and serotonin, suggesting a new physiological mechanism for the pathology. Even single-gene diseases can come with diverse and complex pathologies. Yet, the complexities in this monogenic disease could be understood (35).

With experiences with infectious diseases and inborn errors of metabolism, and inspired by successes in molecular genetics, there was initial hope that cancer might also derive from a mutation in a single gene, and would thereby become understood and curable once that single ‘oncogene’ had been identified. Indeed, overexpression of some genes occasionally correlated with tumorigenesis. However, not just one but more than a hundred such ‘oncogenes’ were found (36), suggesting a much higher complexity than anticipated. Part of this complexity was described by Hanahan and Weinberg in their 2000 report (37) on six ‘hallmarks’ of cancer: proliferative signaling, evasion of growth suppressors, activation of metastasis, replicative immortality, angiogenesis, and resistance to cell death. However, a hallmark is a characteristic, i.e., not the effective cause one was hoping for. Hornberg *et al.* (16) then formulated what was perhaps implicit but surprisingly not made explicit in the Hanahan and Weinberg report (37), i.e., that this complexity derived from the feature that cancer is a ‘systems biology disease’, i.e., a disease caused by faults in the cellular network rather than in a unique molecule. Many different faults in a network can lead to similar changes in network behavior.

In 2011, Hanahan and Weinberg added two hallmarks and two enabling characteristics (38). The former finally included the deregulation of cellular energetics, already long known as the Warburg effect. The enabling characteristics were genome instability/mutation, and inflammation. They were presented as indirect causes. Considering the enhanced mutation rate of most tumors, one may envisage a tumor as a cell lineage with accelerated evolution toward cells that are immune to internal and external brakes on their proliferation and movement. We therefore propose that the hallmarks of cancer correspond to final causes of tumorigenesis. The earliest and fairly universal hallmark of cancer, i.e., the Warburg effect, may well be a good example of this: cells with their own proliferation, rather than the proliferation or persistence of the entire multicellular organism, as objective will outgrow cells with the normal order of priorities. They will develop as proliferating cells with a Warburg effect. This is because the low efficiency of glycolysis, requiring much increased glucose uptake in order to be able to synthesize the same amount of ATP, is not a concern for this asocial cell type. The rest of the body, particularly the liver, will do its utmost to maintain the glucose levels at their normal level, and succeed up to a significant tumor load to the body. Accordingly, the objective of a high growth rate of a minority cell type in a multicellular organism causes it to develop as a tumor cell, unless this is prevented by intensive intercellular signaling (31).

The objective function of FBA is a final rather than efficient cause. Thereby, FBA may be a method to deal with final causation. Recently, FBA was developed further so as to be able to deal with different cell subpopulations competing for common nutrients. Using the final cause (objective function) of maximal growth rate of all cells combined, it was shown that insensitivity to negative cell–cell signaling, rather than an increased inherent growth rate, turned the population into a tumor (31). Here, the cause of tumorigenesis was a final cause, i.e., growth rate.

## Network diseases

**Figure 1:**
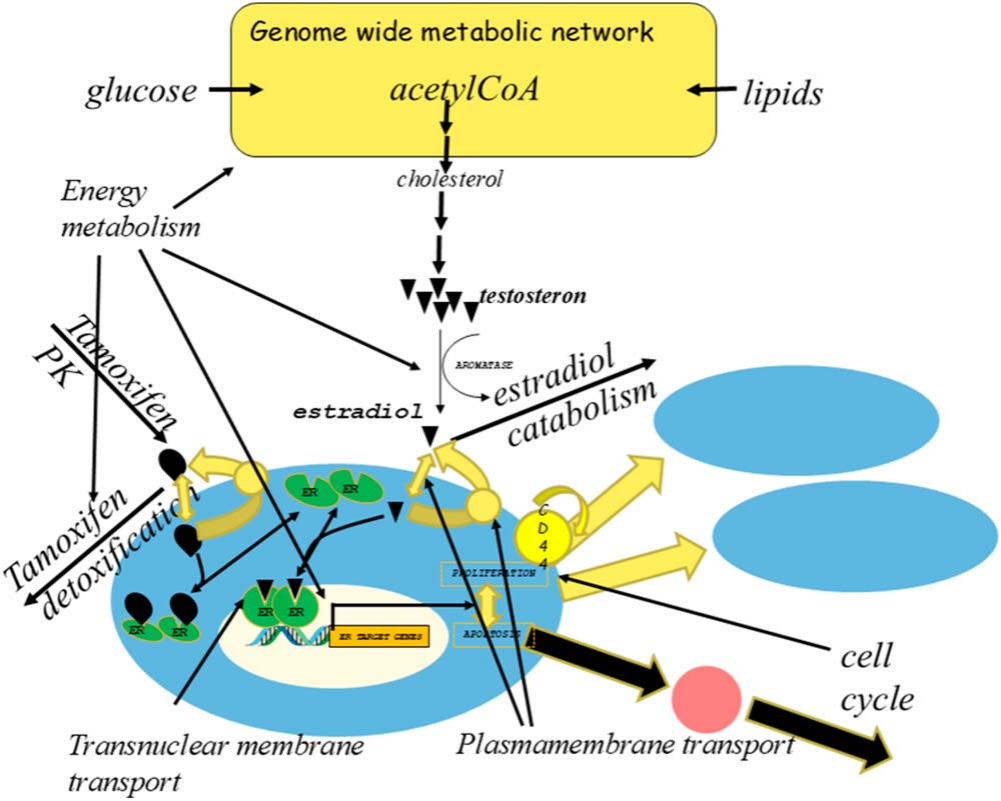
ER signaling as an example of the complexity of important intracellular networks. Depending on the stage in the menstrual cycle, as expressed by the estradiol level in the blood, one of the nuclear hormone receptors, i.e., the ER, binds estradiol and thereby obtains an increased affinity for promoter sites on the nuclear DNA. Its binding then changes the expression level of genes that are controlling the balance between cell proliferation and apoptosis. At high estradiol levels, the cells proliferate, and at low levels they undergo apoptosis. Under physiological conditions this brings about a monthly cycle of growth and reduction of the corresponding breast tissue. Synthesis of estradiol occurs in the ovaries and some of its degradation in the liver. It is made from testosterone, which derives from cholesterol synthesized by the liver or taken up by the intestines. The networks controlling the ER activity are even more complex than depicted, as they include gene expression networks that determine the expression level of the ER and the concentrations of the enzymes and transporters that play a role. They also include signal transduction networks that control the phosphorylation of the alpha subunit of the ER. Breast cancer may occur when the estrogen level in the breast cells is increased or fails to decrease periodically, so that the proliferation genes are more highly or constantly expressed and the apoptosis is repressed. In ER+ breast cancer the cancer cells still have ER, with the implication that the receptor may be inactivated. Such inactivation is achieved by the drug tamoxifen. Pharmacokinetics networks determine the concentration of the drug tamoxifen in the breast. Tamoxifen is transported into the cells and may be extruded or bind to the ER in competition with the estradiol. The tamoxifen-bound ER has a lower affinity for the DNA and is subject to degradation. The drug tamoxifen is highly successful in ER-positive breast cancer, but in some cases drug resistance occurs. Inspection of the network helps understand that there are various mechanisms of drug resistance. One of these is the enhanced expression of the drug efflux pump in the plasma membrane of the cells. Another is mutations in the ER decreasing its affinity for tamoxifen or increasing its affinity for estradiol. And yet another is changes in the pharmacokinetics of tamoxifen.

What is a systems biology disease? What is a network disease? The concept of systems biology disease derives from the realization that a physiological function is usually not provided by a single molecule, but by a network of molecules. From the finding that in such networks multiple enzymes share in the control of flux and concentrations, and thereby pathway function (9, 10), it was inferred that a reduced activity of any of those enzymes would cause the disease. Because inhibition of pathway flux at different points in a pathway has different effects on the metabolome, the different diseases might differ somewhat. Consequently, the diseases should be expected to be multifactorial, both in terms of multiple causative factors and in the sense of diverse and multiple pathologies. Figure 1 illustrates part of the complexity of the networks involved, here for estrogen receptor (ER)-positive breast cancer.

The network organization of cell biology readily explains why Mendelian genetics fails to identify single key genes in most diseases (38, 39). Indeed, the complexity around causation increases when there is redundancy due to multiple pathways leading to the same function (12). Should there be three such pathways, each with ten flux-controlling steps, then three losses of molecular function should be required for the disease to emerge. However, the corresponding three mutations would usually not be in the same three genes, as there should be 1,000 different triplets of mutations that would cause the disease. Every single mutation would only be found in 10% of all the disease cases, and would on its own (due to metabolic compensation) have only a minor, if any, disease effect. This makes it difficult even for a genome-wide association study (GWAS) to detect all genes that are involved in a disease. Multiple genes should be expected to have effects on the disease, but mostly with a low penetrance (low impact in terms of % of people with the mutation contracting the disease). Genes that reside close to the functional endpoint of the pathway may have higher penetrance but still be mutated only at low frequencies in the patient population (because other mutations can affect the same pathway). Indeed, GWAS studies have been costly and perhaps somewhat disappointing in terms of the number of single causative genes they have revealed (e.g., (40)). These did include high-penetrance genes such as BRCA1 and BRCA2 (41), which are responsible for only a few % of all breast cancer cases, however. Moreover, these two genes belong to a different class of mutation, as they are involved in repair such that their deletion enhances the mutation rate. Other genes such as MYC are high up in the hierarchy of regulation of cell function and thereby had an increased probability of being detected (38).

More successful than the initial focus on single genes has been the identification of polygenic risk scores (and causes) for breast and prostate cancer (e.g., (42)). A GWAS success story, Crohn’s disease, indeed appears to be due to a cumulation of weak effects of a great many genes (43). It has indeed helped to consider systems not as collections of molecules, but as collections of pathways. Gene Set Enrichment Analysis (GSEA), with the set referring to predefined pathways or subnetworks (44) have met with success. Polygenic risk scores are also being analyzed from a pathway point of view (e.g., (45, 46)) rather than as sets of functionally unrelated genes.

In a systematic approach enhanced by modeling, one might assign a person with a particular single-nucleotide polymorphism (SNP) to bins corresponding to pathways in which the corresponding gene product plays a potentially controlling role. This assignment should not be limited to supposedly rate-limiting steps, as metabolic control analysis has shown that flux limitation is shared by multiple enzymes in a pathway (10, 21). Moreover, since most enzymes play roles in multiple pathways, each person with an SNP should be assigned to all these pathway bins. The ‘pathways’ may be defined by physiological functions of potential importance for the particular disease (such as glucose metabolism, lactate metabolism, and ROS production). It should then be examined whether a person has SNPs in all pathways leading to any functionality important for a certain disease. These persons should be correlated with the occurrence of the disease in them.

Bayesian statistical analysis should then help identify individuals with increased disease propensity (47). Alternatively, one should construct a comprehensive mathematical model of the functionalities affected in the disease as functions of the activities of all the genes. Genes with an SNP should then be knocked out in that model, and the effect on the functionalities evaluated by computation. The computed effects on the functionalities of persons should then be correlated to the disease observed in those persons. In this approach, sufficient statistical power should be achieved with relatively small study populations, so that this MAGWPAS (Modelling Assisted Genome-Wide Pathway Association Study) might become more successful than GWAS and even GSEA have been. For some metabolic diseases, such a MAGWPAS approach should already be feasible, as gene-wide metabolic maps are available and FBA does not require the kinetic details that are harder to measure experimentally. The problem with diseases other than metabolic diseases is that most of their pathology may derive from faults not in metabolism itself but in the regulation thereof (MYC constituting an example), which is not comprised in the usual genome-wide metabolic maps (26). Maps taking regulation (through signal transduction and gene expression) into account are much more difficult to make, as they require much more quantitative experimental information and are not constricted by conservation of chemical elements across their processes.

In anticipation of this new approach, one may wonder whether it helps to realize that a particular disease is a network rather than a single-gene disease. One advantage of realizing that a disease is a systems biology disease is the concomitant appreciation that there will be multiple simultaneous causes, i.e., multiple genes or nutritional components need to be affected at the same time, corresponding to multiple pathways being affected. Moreover, one will realize that each pathway may have been compromised in multiple ways. We call this an AND + OR situation; e.g., pathway A, pathway B, AND pathway D should all three be affected, whereas in pathway A (or B, or C) it could be gene 1, gene 2, OR gene 3 if it is a three-gene pathway. The disease is thus multifactorial in two different ways, which adds much to the complexity already exemplified above. An immediate implication is that hitting a single target with a single drug will rarely suffice to eliminate the disease. Because most drugs do not take out their target by 100%, the substrate of their target will increase in concentration, thereby reducing the effectiveness of the drug, especially if the drug is a competitive inhibitor (23), if there are parallel pathways (12), or if there is a parallel pathway that is not inhibited and may be induced further by the substrate. The increase in concentration mentioned occurs naturally in metabolic pathways, but should also be expected in signal transduction pathways through homeostatic feedback loops. Due to the positive feedback loops in much paracellular signaling possibly including the androgen receptor (AR) (48), the system may be in one of a set of distinct stable states, and it should be difficult to attain the extent of inhibition required to move the system away from that state.

When disease is caused by overexpression of a gene, one might be inclined to add drugs that reduce the expression level. However, the overexpression causes the process to be less controlling, so that the inhibitor of that very step should not work well. It might then be better to add drugs that affect other steps in the pathway that have become more controlling due to the overexpression (16). In cancer, this consideration may be overruled by even more complexity. Overexpression of a gene that has a tumorigenic effect will also affect other functions. As the physiological functions are close to optimal due to long biological evolution, these additional effects are often negative. This creates selection pressure for secondary mutations that reoptimize these other functions. Removing the effect of the overexpression in the persistence of these secondary mutations then causes this secondary function to be suboptimal. Through the secondary mutations, the network has become ‘addicted’ to the original overexpression. In this more complex case, inhibition of the originally overexpressed gene might still help to combat the disease. These complexities call for pretesting, monitoring, and modeling the effects of anticancer drugs. In view of the principle of shifts in what controls the flux, one may further consider a strategy known from the martial art *Judo*: add an inhibitor that shifts the control to a different step in the pathway, and then add an inhibitor of the latter step. All these complexities of networks around disease causes and drug targets (e.g., Fig. 1), are overwhelming. This calls for systems biology models that comprise all the known regulatory effects and then serve as aids to human intuition. These models can be as ‘simple’ as cartoons, or may be elaborated into more quantitative mathematical models, such as those that may help manage inflammation (e.g., (49)).

Individual humans will differ in terms of their genotype; they have different SNPs in various pathways. Another aspect of the network structure is that those SNPs may still affect the functioning of the pathway directly relevant for the disease. Diseases, but also the effects of therapies, will thereby differ between individual patients. Even within a tissue of a single patient, somatic mutations and different local circumstances will have made the cell population heterogeneous. This is even worse in tumors, with their often increased frequency of mutagenesis. No two patients, and not even two tumor cells within a patient, will be the same. This will not only reduce the effectiveness of drugs, it will also create resistance against drugs that aim to reduce cell viability or proliferation. Cells that are less sensitive to the drug will outgrow the cells that are sensitive, so that the drug’s effect will decrease over time. The heterogeneity of cell populations has been much assessed by single-cell transcriptome sequencing. Since most mRNAs have shorter lifetimes than the corresponding proteins, the transcriptomes at any one point in time may not be representative of the catalytic potential of the individual cells, however (Y Liu and H V Westerhoff, unpublished observations). Single-cell proteomics may be required to assess the most relevant aspects of cell–cell heterogeneity in cell populations.

Nuclear hormone receptors such as the ER or the AR are parts of such complex networks (see Fig. 1). The synthesis of the hormones estradiol and testosterone is accomplished by a metabolic pathway that involves various organs, even down to the liver and intestinal epithelium enhancing cholesterol levels. The ER and AR may each be in one of a set of bistable states managed through positive feedback loops, such as through TGFβ (48). This is indicated in Fig. 1 as the decision between proliferation and apoptosis. Around the stable point of such states, there are homeostatic mechanisms that ensure state stability and thereby incapacitate interference by inhibitors. The proliferative state engages in the expression of multiple genes that are required for cell cycling and biosynthesis. Activated by NCOR2 (a nuclear receptor corepressor) binding, AR leads to deacetylation of histone marks. On the other hand, NCOR2 triggers CpG methylation, which may also impact transcription. The finding that reduced NCOR2 expression accelerates prostate cancer recurrence upon androgen deprivation therapy (50) suggests that NCOR2 binding of androgen-bound AR limits cell proliferation. This in itself appears to work through multiple regulatory routes (50). The degradation pathway of the receptor proteins that can be activated by compounds such as tamoxifen may already be active in the absence of these ligands. Removal of the receptor may thereby be followed by synthesis of new receptor, or continued receptor accumulation in the nucleus. The intracellular levels of estradiol or testosterone are a function of the passive permeability of the plasma membrane and the activity and specificity of the xenobiotic efflux pumps. By itself, cell division will engage a negative feedback loop, as it reduces the concentration of the nuclear hormone receptors. It should be promising to map all processes of potential importance in a diagram more comprehensive than Fig. 1, and then examine how multiple processes may be modulated simultaneously in order to obtain an optimal effect in terms of reduced cell proliferation and increased apoptosis or differentiation.

## Self-organizing molecular neural networks (MNNs)?

The systems biology analysis of the molecules of living cells has confirmed that the molecules occur in complex networks. These networks are hierarchical: networks at one level (e.g., metabolic networks) are regulated by networks at different levels (e.g., gene expression networks). The latter networks are again regulated by the former. With information about the network’s environment (e.g., the blood) being received by metabolic enzymes at the beginning of pathways and by receptors that inform signal transduction and gene expression, the total network is able to reorganize itself not only in response to changes in the information it receives from its outside world, but also in response to changes in network components, such as due to mutations or added inhibitors.

During biological evolution and in part even during the lifetime of the individual (or its tumor), the network has been trained to bring about the optimal self-reorganization with respect to environmental and other challenges. Since these challenges have been manifold, the network will appear to respond intelligently to a great many external and internal challenges (18, 24). Responses to a new challenge will be better and more intelligent if the network has been subject to similar challenges during biological evolution. Here an example may be the consideration of reducing glucose in the diet from the point of view that tumors tend to favor the conversion of glucose to lactate for their provision of Gibbs energy (ATP) (51). Starvation of nutrients such as glucose will have occurred frequently during evolution. Consequently, one should expect both normal and tumor cells to have intelligent responses to glucose starvation so that the starvation may not be quite as effective as one would have hoped.

The self-organization of the cell’s networks corresponds to the plasticity of a highly complex network. Signs are that this training has been quite successful for many organisms and cell types existing today. It is often less clear to what extent challenges that occur today are similar to the challenges that have trained the networks during their evolutionary history. If the challenges are similar, however, then the details of the actual organization of the molecular networks may be of less importance, just like the details of the operation of a car matter little if one turns the steering wheel to take a right turn. The situation resembles that of the artificial neural networks that are becoming abundant in many applications today. Their inner mechanisms are hardly important: what is more important is their training sets.

It is unclear at this moment where we are with the networks inside human cells: are they close to simpler networks that can still be understood by manageable mechanistic (silicon cell) models and genome-wide metabolic maps, together with precise experimental determination of molecular mechanisms and their parameters? Or are they molecular neural networks (MNNs) in that they are closer to artificial neural networks, where different individuals or different individual cells within a single tumor may achieve the same functionality through many different (re)organizations of the network? Now is perhaps the time and age of experimental analyses that test whether the mechanistic or this MNN concept is more realistic. Here it should be important to quantify the extent of (self-)organization of these networks, which may start from quantifying their complexity (20).

## MNNs: implications for engineering and therapy

In cases where the mechanistic paradigm is most appropriate, such as with simpler organisms, *in vitro* systems, and infectious or monogenic diseases, one may continue to use systems biology modeling to predict which changes in the enzymes should produce the desired change at the functional level. One may for instance determine which enzyme exerts the highest control over the desired flux and then overexpress the corresponding gene.

In cases where the networks function more like highly complex MNNs, such as perhaps in human cells, one may rather allow mutagenesis or adaptive changes while bringing about conditions that select for the least pathogenic cell types. This corresponds to the training of the MNN. An example of this is a continuous diet that selects liver cell adaptations away from obesity and type 2 diabetes.

Time will tell, and it will do so sooner rather than later provided that we now begin with examining where we are with relevant diseases: a mechanistic or an MNN reality?

## Declaration of interest

The author (HVW) declares that there is no conflict of interest that could be perceived as prejudicing the impartiality of the research reported.

## Funding

This work did not receive any specific grant from any funding agency in the public, commercial, or not-for-profit sector.
